# Integration of Vent Line Monitoring in the Right Superior Pulmonary Vein for Early Detection of Paravalvular Leaks in Aortic Valve Replacement

**DOI:** 10.7759/cureus.84049

**Published:** 2025-05-13

**Authors:** Ignazio Condello, Youssef El Dsouki

**Affiliations:** 1 Cardiac Surgery, University of Insubria, Varese, ITA; 2 Cardiovascular Surgery, Sorbonne Université, Paris, FRA

**Keywords:** aortic valve replacement, cardiopulmonary bypass, paravalvular leak, transesophageal echocardiography, vent line monitoring

## Abstract

This article presents an innovative surgical technique for the early detection, management, and quantification of paravalvular leaks (PVLs) during aortic valve replacement. The method utilizes an endo-cavitary vent in the right superior pulmonary vein to monitor blood flow and oxygen saturation, which is crucial for the early identification and quantification of PVLs. Complementing this, transesophageal echocardiography (TEE) is used for real-time imaging and detailed assessment of the regurgitant volume, providing a comprehensive evaluation of leak severity. This integrated monitoring approach allows for precise and immediate surgical adjustments, aiming to minimize cardiopulmonary bypass (CPB) time and reduce postoperative complications. The technique not only enhances patient safety but also improves surgical outcomes by enabling better management of the surgical process. This article delineates the procedural steps and underscores the benefits of this multifaceted monitoring strategy, outlining its significant implications for advancing clinical practices in cardiac surgery.

## Introduction

Paravalvular leaks (PVLs) present considerable challenges during aortic valve replacement surgeries, with traditional monitoring methods often failing to promptly and accurately detect and quantify these leaks [1,2]. This delay can lead to extended cardiopulmonary bypass (CPB) times and increased postoperative complications, adversely affecting patient outcomes. To address these limitations, our proposed technique integrates vent line monitoring with transesophageal echocardiography (TEE) to establish a real-time detection and evaluation system, and hence can be called TEE-V. TEE is the gold standard to detect volume in the heart cavity in extracorporeal membrane oxygenation [3] and extracorporeal technologies. This system not only detects PVLs more rapidly but also quantifies the regurgitant volume. In aortic regurgitation, regurgitant volume refers to the amount of blood flowing back into the left ventricle from the aorta during diastole. A regurgitant volume of 60 mL/beat or more and a regurgitant fraction (RF) of 50% or more are considered indicative of severe aortic regurgitation, providing critical data that informs immediate and targeted surgical corrections [4]. By improving the accuracy of both detection and quantification of PVLs, this approach substantially reduces CPB duration and enhances the effectiveness of interventions, thereby improving patient recovery and setting new benchmarks for cardiac surgical care.

## Technical report

Description of the technique

**Figure 1:**
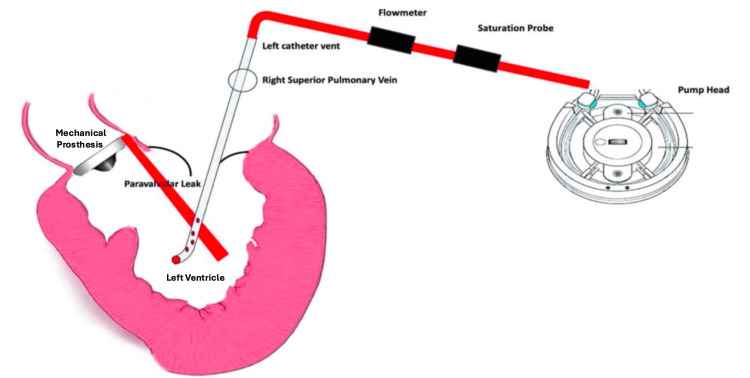
Vent line monitoring in the right superior pulmonary vein for early detection of paravalvular leaks in aortic valve replacement. Image Credit: Ignazio Condello and Youssef El Dsouki

No formal patient consent was required for this study, as it involves a theoretical and practical description of the technique without application to patients. The procedure consists of the strategic placement of an endo-cavitary vent in the right superior pulmonary vein, with its positioning confirmed intraoperatively through imaging techniques to ensure optimal setup. The vent line is equipped with a flow meter and an oxygen saturation detector, both calibrated preoperatively to guarantee measurement accuracy (Figure 1). Throughout the simulated procedure, real-time data is displayed to the surgical team, focusing on critical parameters such as abnormal flow rates and oxygen saturation levels after cross-clamp removal. In particular, the detection of a continuous abnormal flow of oxygenated blood returning through the pulmonary vein vent following cross-clamp removal could theoretically suggest the presence of a PVL. Upon observing such a finding, the perfusionist would promptly alert both the surgeon and the anesthesiologist, enabling immediate TEE assessment to confirm the diagnosis and guide further intraoperative management.

Application of the technique

The application described remains at the theoretical and simulation level. To maximize the potential effectiveness of the vent line monitoring system, preoperative procedures such as equipment testing and team training in interpreting monitoring data are emphasized. Particular focus is placed on the phase immediately before and after de-airing, where the interpretation of real-time vent line data, especially the presence of continuous abnormal oxygenated blood flow, could alert the team to a potential PVL. This assessment will be further supported by a pulse oximeter placed on the vent line to continuously sense oxygen saturation. In this conceptual model, any anomalies detected would prompt the perfusionist to immediately notify the surgical and anesthetic teams, with subsequent confirmation by TEE [5,6]. This would allow for timely corrective action, theoretically minimizing time on CPB and improving patient outcomes. However, no clinical application on actual patients was performed during the study phase.

## Discussion

The integration of endo-cavitary vent monitoring with TEE represents a significant theoretical advancement in the intraoperative management of PVL during aortic valve replacement surgeries. Conceptually, this combined approach could enable earlier detection and more accurate quantification of PVLs, both of which are critical for the prompt implementation of corrective strategies [2]. The majority of patients are asymptomatic; only 1-5% present with heart failure and/or hemolytic anemia. The indications for re-intervention are not yet universally accepted. The severity of symptoms and degree of paravalvular regurgitation (moderate to severe) are accepted as indications for intervention. Medical management of heart failure and hemolytic anemia with red blood cell transfusion, erythropoietin, intravenous iron, or folic acid is of limited value. Traditionally, a surgical approach with a new reintervention valve replacement has been the accepted treatment. However, reoperation is associated with higher mortality and morbidity than the initial operation, with reported mortality ranging from 6% to 42% and important morbidity, which includes, among others, perioperative stroke (5.1%), sternotomy wound infections and reconstruction (25%), long hospital stays, and recurrence of the leak in the same place in the range of 20-37%. For these reasons, patients with PVLs are considered high-surgical-risk or inoperable, and percutaneous techniques have been developed in an attempt to repair them [3,4]. Traditionally, intraoperative PVL diagnosis has relied primarily on echocardiographic assessment, which, although effective, can be limited by operator dependency and interpretative variability [3]. The addition of vent line monitoring introduces an additional layer of safety by theoretically allowing the identification of hemodynamic anomalies, such as abnormal drainage flow and oxygen saturation levels, that may serve as early indicators of a potential PVL, sometimes even before it becomes fully apparent on echocardiography. Particularly significant is the potential role of monitoring drainage parameters, including pulmonary venous drainage pressures and oxygen saturation, in suggesting both the presence and severity of a PVL. In the proposed model, an abnormal, continuous return of oxygenated blood through the pulmonary vein vent following cross-clamp removal could prompt immediate attention. This signal would theoretically alert the perfusionist, who would then notify the surgical and anesthetic teams, leading to timely verification and diagnosis through TEE [4]. The theoretical integration of vent monitoring with echocardiographic imaging could allow for reduced intraoperative decision-making times, minimized CPB duration, and facilitated timely interventions, such as additional suturing or prosthesis repositioning, potentially preventing the development of more serious complications. Importantly, the proposed system is designed to be safe and easily integrated into existing surgical workflows without introducing significant additional risks. Nevertheless, while this conceptual framework highlights potential advantages, further experimental validation and clinical studies are necessary to confirm its real-world applicability and impact on patient outcomes, including morbidity, mortality, and hospitalization duration [5,6]. In addition, the development of dedicated instrumentation specifically designed for real-time flow and oxygen saturation monitoring in this context will be crucial to fully realize the potential of this technique [7]. Future improvements could include the automation of monitoring systems to further enhance the precision, reliability, and ease of interpretation by the surgical team.

## Conclusions

The conceptual integration of endo-cavitary vent line monitoring with TEE offers a promising strategy for enhancing the intraoperative detection and management of PVL during aortic valve replacement procedures. The combination of continuous real-time hemodynamic measurements with dynamic imaging could theoretically improve the accuracy and timeliness of intraoperative assessments, supporting immediate and more targeted surgical interventions when necessary. This dual-monitoring approach has the potential to minimize the reliance on subjective interpretation alone, offering an additional layer of intraoperative control that may contribute to reducing CPB times and improving postoperative outcomes. Furthermore, early identification of hemodynamic anomalies could facilitate the prevention of significant complications associated with undiagnosed PVLs. Although these insights are based on a theoretical model and simulated conditions, they highlight important areas for future investigation. Validation through clinical studies will be essential to confirm the practical benefits of this technique. In parallel, the development of specialized equipment tailored for integrated vent line and TEE monitoring could further optimize its application and broaden its use in routine surgical practice.
